# Role of P2X_7_R in the development and progression of pulmonary hypertension

**DOI:** 10.1186/s12931-017-0603-0

**Published:** 2017-06-24

**Authors:** Jie Yin, Shuling You, Haopeng Liu, Li Chen, Chengdong Zhang, Hesheng Hu, Mei Xue, Wenjuan Cheng, Ye Wang, Xinran Li, Yugen Shi, Nannan Li, Suhua Yan, Xiaolu Li

**Affiliations:** 1 Department of Cardiology, Shandong Provincial Qianfoshan Hospital, Shandong University, No. 16766 Jingshi Road, Lixia District, Jinan, Shandong Province China; 2 Adicon Company, Department of Pathology, Wangkai Infectious Diseases Hospital of Zaozhuang City, Zaozhuang, Shandong Province China; 3 Department of Neurosurgery, Zhangqiu People Hospital, Jinan, Shandong China; 4 Department of Gastroenterology, the Affiliated Hospital of Qingdao University, Qingdao, China; 5 Department of Orthopedics, the Affiliated Hospital of Qingdao University, Qingdao, China; 6 Department of Emergency, Shandong Provincial Qianfoshan Hospital, Shandong University of Traditional Chinese Medicine, No. 16766 Jingshi Road, Lixia District, Jinan, Shandong Province China; 7 Department of Emergency, Shandong Provincial Qianfoshan Hospital, Shandong University, Jinan, China

**Keywords:** Pulmonary hypertension, P2X_7_R, NLRP3, Macrophage

## Abstract

**Background:**

Pulmonary arterial hypertension (PAH) is a devastating disease that lacks sufficient treatment. Studies have shown that the Nod-like receptor family, pyrin domain containing 3 (NLRP3) inflammasome contributes to PAH pathogenesis, but the role of the upstream molecular P2X_7_ receptor (P2X_7_R) has remained unexplored. We investigated the role of P2X7R in the pathogenesis of PAH.

**Methods and results:**

PH was induced by a single subcutaneous injection of monocrotaline (MCT) (60 mg/kg) on left pneumonectomised Sprague-Dawley rats, as validated by significant increases in pulmonary artery pressure and vessel wall thickness. Marked P2X_7_R was detected by predominant PA immunostaining in lungs from PH rats. Western blot revealed a significant increase in the protein levels of P2X_7_R as well as NLRP3 and caspase-1 in the diseased lung tissue compared with normal tissue. The rats received A-740003 (a selective P2X_7_ receptor antagonist, 30 mg/kg) daily starting from 1 week before or 2 weeks after MCT injection. Consequently, A-740003 reversed the NLRP3 inflammasome upregulation, significantly decreased the mean right ventricular (RV) pressure and RV hypertrophy, and reversed pulmonary arterial remodelling 4 weeks after MCT injection, as both a pretreatment and rescue intervention. Notably, A-740003 significantly reduced macrophage and pro-inflammatory cytokine levels, as measured via bronchoalveolar lavage. The recruitment of macrophages as well as collagen fibre deposition in the perivascular areas were also reduced, as confirmed by histological staining.

**Conclusions:**

P2X_7_R contributes to the pathogenesis of PH, probably in association with activation of the NLRP3 inflammasome. Blockade of P2X7R might be applied as a novel therapeutic approach for the treatment of PAH.

**Electronic supplementary material:**

The online version of this article (doi:10.1186/s12931-017-0603-0) contains supplementary material, which is available to authorized users.

## Background

Pulmonary arterial hypertension (PAH) is a rare but life-threatening disease characterised by pulmonary vasoconstriction, endothelial cell proliferation, smooth muscle cell proliferation, and *in situ* thrombosis, leading to progressive pulmonary hypertension and ultimately causing right ventricular (RV) failure and death [1–3]. The current therapies licensed for PAH focus on vasodilation [4]. Drugs targeting the prostacyclin, endothelin-1 receptor, and phosphodiesterase pathways improve symptoms and exercise tolerance, but persistent morbidity and mortality indicate that important pathogenic mechanisms are minimally affected [5, 6]. There is accumulating evidence of a specific contribution of NLRP3 and related inflammasomes, and their regulated cytokines or receptors may represent novel diagnostic or therapeutic targets in pulmonary diseases, including PAH [7–9].

The NLRP3 inflammasome comprising the apoptosis speck-like protein containing a caspase-recruitment domain (ASC), NLRP3, and procaspase-1, plays a key role in innate immunity and lung injury [10]. The NLRP3 inflammasome is activated in response to cellular stresses through a two-component pathway involving a Toll-like receptor 4-ligand interaction (priming) followed by a second signal. In particular, extracellular ATP is the best-known danger signal in NLRP3 activation via stimulation of the P2X_7_ purinergic receptor (P2X_7_R) [11]. Despite acting as a co‑stimulus or second signal for the formation of an NLRP3 inflammasome, the role of P2X_7_R has not been previously characterised in models of PH. P2X_7_R is a highly unusual ATP-gated non-selective cation channel expressed primarily on cells of haematopoietic origin, such as macrophages and microglia. P2X_7_R signalling is involved in the regulation of many physiological and pathophysiological processes such as silica-induced lung-disease. High extracellular ATP levels are released into the extracellular medium due to cell damage, hypoxia or mechanical stress, alerting the immune system to sites of cell damage/injury [12]. Recently, there has been growing evidence to implicate the ATP-P2X7-inflammasome-caspase 1-IL-1/18 axis in lung diseases such as murine models of hyperoxia-induced acute lung injury, smoke-induced airway inflammation and patients suffering from COPD [13, 14]. P2X_7_R, based on its role in the processing of the NLRP3 inflammasome and IL-1β, represents a reasonable target in the study of the pathogenesis of PAH.

Therefore, the purpose of this study was to determine the extent to which the inhibition of P2X_7_R would suppress pulmonary vascular remodelling in an animal model with neointimal lesions resembling the neointimal lesions found in PAH. For pharmacological P2X_7_R inhibition, we applied A-740003, which is a competitive antagonist of P2X_7_R and is more potent and selective than any other antagonist with fewer species-dependent differences in various preclinical disease models [12, 15, 16].

## Methods

### Animal models

Male Sprague-Dawley rats (weighing 300–330 g, obtained from the Laboratory Animal Center, Chinese Academy of Science, Beijing) were used in this experiment. The rats were housed at 20 ± 3 °C under a 12-h light/12-h dark cycle with free access to food and water. All procedures were conducted according to approved protocols and guidelines established by the Shandong University Institutional Animal Care and Use Committee.

Rats were randomly assigned to one of four possible groups: group A (*n* = 15) was the sham group; group B (*n* = 30) was the P/MCT group, in which PH rats received vehicle treatment; group C (*n* = 20) was the P/MCT + CA group, in which PH rats received continuous and early administration of A-740003; and group D (*n* = 20) was the P/MCT + DA group, in which PH rats received delayed administration of A-740003. PH was induced by left pneumonectomy plus MCT injection. For anaesthesia, the animals received 2% xylazine (4 mg/kg)/ketamine (100 mg/kg) and were intubated. After connection to a small-animal ventilator (HX-300S, TME, Chengdu, China), the animals received an adjusted rate of 60 breaths/min and a tidal volume set to 1.1–1.3 mL/100 g body weight, followed by a left unilateral pneumonectomy as described previously [17]. MCT (Sigma, St. Louis, MO) was prepared as previously described. MCT (60 mg/kg) was injected subcutaneously 1 week later. All animals were monitored daily until they developed pulmonary hypertension symptoms such as weight loss and tachypnea.

Animals in Group C received continuous and early A-740003 (Sigma-Aldrich), a selective P2X_7_R inhibitor, intraperitoneally at a dose of 50 mg/kg from the time of pneumonectomy to day 28 [18–20]. Animals in Group D received delayed administration of A-740003 drug 2 weeks before harvest (2 weeks after MCT). Once initiated, A-740003 in Group D was continued until harvest. Sham control animals received water alone. Haemodynamic, morphologic, and biochemical assessments were performed on day 28 after MCT injection.

### Echocardiography and haemodynamic measurements

The rats in the experimental groups were anaesthetised by intraperitoneal injection of sodium pentobarbital (30 mg/kg). The room temperature was maintained at approximately 25 °C. A Visual Sonics Vevo 770 echocardiographic machine (Visual Sonics, Toronto, Canada) equipped with a 14-MHz linear transducer was used to assess cardiac function. The measurements were performed in a blinded manner by an echocardiographic expert. Short- and long-axis B-dimensional parasternal views of both ventricles at the level of the papillary muscles were acquired to visualise the areas of the left ventricle (LV) and the right ventricle (RV). Cardiac output and stroke volume were obtained from the B-mode long axis according to Simpson’s method, while the pulmonary artery diameter and RV wall thickness were obtained in M-mode. Doppler was applied to the pulmonary artery to obtain the pulmonary artery acceleration time [21].

Blood pressure was evaluated with the tail-cuff method using a non-invasive automatic blood pressure recorder (BP-98A; Softron, Tokyo, Japan). Each value was the average of at least three consecutive measurements [22]. Prior to sacrifice of the animals, RV systolic pressure (RVSP) was transduced from the right jugular vein into the vena cava and then into the right atrium followed by the right ventricle using a 1.4 F Millar Mikro-Tip catheter transducer (Millar Instruments Inc., Houston, TX). The position of the catheter into the right ventricle was validated by an acutely increased pressure wave accompanied by the loss of resistance, and RVSP was then measured with Power Lab monitoring equipment (Millar Instruments). Haemodynamic values were automatically calculated using a LabChart 7.0 physiological data acquisition system (AD Instruments, Sydney, Australia). The animals were then euthanised prior to sacrifice.

### Tissue processing and histology

After acquiring the above measurements, cardiac arrest was induced by injection of 2 mmol KCl through the catheter. The left lungs were then weighed. The lung was then separated longitudinally into two parts: one was removed and frozen in liquid nitrogen for western blot analysis, and the other was inflated with 0.5% low-melting agarose at a constant pressure of 25 cm H_2_O, fixed in 10% formalin for 24 h and used for small pulmonary artery and IHC analyses. Next, the heart was excised, and the weight ratio of the right ventricle to the left ventricle plus the septum (RV/LV + S) was determined using Fulton’s index [23].

### Western blot

For immunoblot analyses, modified RIPA buffer (Beyotime Institute of Biotechnology, Jiangsu, China) was used to extract total protein from frozen lung tissue [24]. Extraction proteins from tissues and cells were measured using the BCA protein assay reagent kit (Pierce). An equal amount of total protein (80 μg of protein/lane) was resolved on a 5–12% SDS-PAGE gel and transferred onto a polyvinylidene difluoride (PVDF) membrane. The membranes were blocked with 5% nonfat dry milk in PBST (containing 0.05% Tween 20). Incubation with the antibodies was performed using the following dilutions: 1:750 for P2X_7_R (Abcam, USA) and 1:1000 for caspase-1, procaspase-1, caspase-1, IL-1β (all of them from Cell Signaling Technology, USA) and total NLRP3 (Biosource, Belgium). Primary antibodies were detected with horseradish peroxidase-conjugated antibodies, 1:5000 for anti-mouse (ZSJQ-BIO, Beijing, China) and 1:5000 for anti-rabbit (ZSJQ-BIO, Beijing, China), at room temperature for 1.5 h. Blots were developed using an enhanced chemiluminescence (ECL) detection kit (Millipore) and visualised using a FluroChem E Imager (Protein-Simple, Santa Clara, CA, USA). Blot bands were qualified using NIH ImageJ software.

### RT-PCR

Total RNA was extracted from lung tissues with TRIzol reagent (Invitrogen). cDNA was synthesised from 2 μg RNA using a Prime Script RT Reagent Kit (TaKaRa, Dalian, China) as described previously. mRNA expression was determined using gene-specific primers and SYBR Green 1 with a Bio-Rad iQ5 Multicolor Real-Time PCR machine (Bio-Rad Laboratories). For each sample, both GAPDH and the target gene were amplified in triplicate in separate tubes. The relative gene expression was calculated using the 2^-ΔΔCT^ method [25] and normalised to GAPDH expression. The primers used in this study were as follows:

NLRP3: forward, 5′-CTGCATGCCGTATCTGGTTG-3′, reverse, 5′-GCTGAGCAAGCTAAAGGCTTC-3′;

Caspase-1: forward, 5’-ACTCG TACAC GTCTTGCCCTCA-3’, reverse, 5’-CTGGGCAGGCAGCAAATTC-3’;

P2X_7_R: forward, 5′-CTACTCTTCGGTGGGGGCTT 3′, reverse, 5′- AACCCTGGTCAGAATGGCAC 3′;

IL-1β: forward, 5′-GCACAGTTCCCCAACTGGTA-3′, reverse, 5′-TGTCCCGACCATTGCTGTTT-3′;

GAPDH: forward, 5′-AGATCCACAACGGATACATT-3′, reverse, 5′-TCCCTCAAGATTGTCAGCAA-3′;

### Immunohistochemistry

The left lung lobes were longitudinally cut and processed as described previously [21] by preparing standard formalin-fixed, paraffin-embedded tissues for HE or regular immunohistochemistry staining. Tissue samples were sectioned at a thickness of 5 μm [26]. In each lung section, 30 small PAs (50–100 μm in diameter) were analysed at × 40 in a blinded manner. The medial wall thickness was expressed as the summation of two points of medial thickness/external diameter × 100 (%). Intra-acinar (precapillary) PAs (20–30 μm in diameter, 25 vessels each) were assessed for occlusive lesions as Grade 0 for no evidence of neointimal lesion, Grade 1 for less than 50% luminal occlusion, and Grade 2 for more than 50% luminal occlusion [27]. There was no evidence of neointimal lesion formation in any PAs from normal rats (all PAs were graded as 0). Fibrosis was evaluated on heart tissue sections stained with Masson’s trichrome (Jiancheng, China) according to standard protocols [28]. Anti-P2X_7_R (1:100, Abcam), α-SMA (1:500; Abcam) and anti-CD68 (1:150; Abcam) antibodies were used as primary antibodies. Subsequently, slides were incubated using an ABC Elite Kit (Vector Laboratories) and DAB substrate (Vector Laboratories) and counterstained with haematoxylin.

For immunofluorescence, samples were incubated with anti-P2X_7_R antibody (1:50; Abcam) and α-SMA (1:200; Abcam) or FITC-CD68 (1:100; Abcam) overnight at 4 °C, followed by a 2-h incubation with Alexa 545-conjugated goat anti-rabbit (1:100; Peprotech) or FITC-conjugated goat anti-rabbit (1:200; Abcam) and FITC-conjugated rabbit anti-mouse (1:200; BioLegend) secondary antibodies. The α-SMA -positive cells in each group were double-immunostained with anti-proliferating cell nuclear antigen (PCNA) (1:300; Abcam). The sections were counterstained with DAPI (Life Technologies) to identify nuclei. The contribution of α-SMA to PCNA expression was measured semi-quantitatively by the proportion of colocalization cells (i.e. yellow staining in merged images) divided by the number of corresponding staining SMA cells.

A pathologist blinded to the study reviewed 10 sections per lung. All images were obtained using an Olympus LCX100 Imaging System and analysed with ImageJ software (version 1.38x; National Institutes of Health). The Institutional Review Board of Shandong University approved the studies.

### Bronchoalveolar lavage fluid (BALF)

Bronchoalveolar lavage was collected and analysed for macrophage influx and cytokines such as TNF-α and IL1-β as described previously [29]. Briefly, the trachea was cannulated, and BALF was obtained by administering three consecutive injections of phosphate-buffered saline (PBS) to a final volume of 1.0 mL. The BALF was then centrifuged at 400 *g* for 10 min (Mikro 22 R, Hettich), and the supernatant was stored at −20 °C. A double-antibody sandwich enzyme-linked immunosorbent assay (ELISA) kit (Peprotech, NJ) was used to detect serum TNF-α and IL-1β concentrations according to the manufacturer’s instructions. The intra- and inter-sample variability for each kit was less than 8%.

### Statistics

The data are presented as the mean ± standard deviation (SD). The unpaired *t*-test was used to compare values between two groups. Analysis of variance (ANOVA) was used to compare differences among more than two groups, followed by a Newman-Keuls test. Analyses were performed using SPSS 17.0 software (SPSS Inc. Chicago, IL, USA). A *p*-value < 0.05 was considered statistically significant.

## Result

### Increased expression of P2X_7_R in lungs from PH rats with pulmonary hypertension

**Fig. 1:**
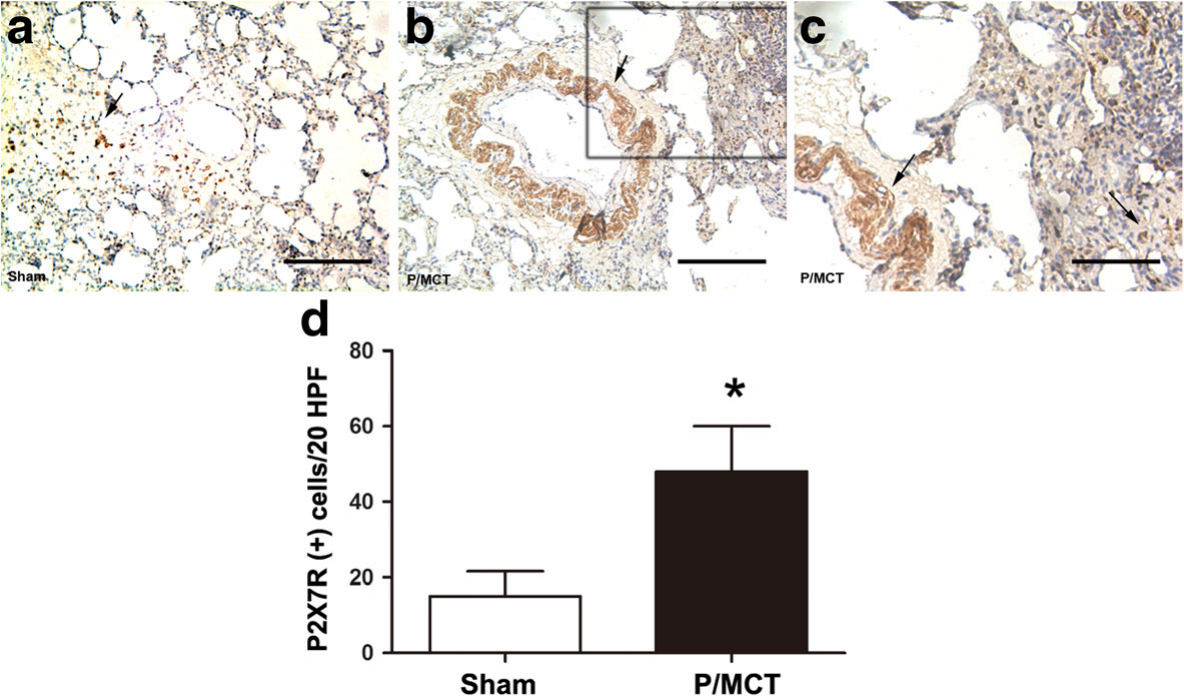
IHC staining of P2X_7_R in the sham group (**a**) and P/MCT group (**b**, **c**) at 4 weeks after MCT injection. Quantification of P2X7R-positive cells per 20 high-power fields (HPFs) (**d**). Original magnification × 20. *Scale bar* = 50 μm for all images. IHC: Immunohistochemistry; P/MCT: MCT plus left pneumonectomy

**Fig. 2:**
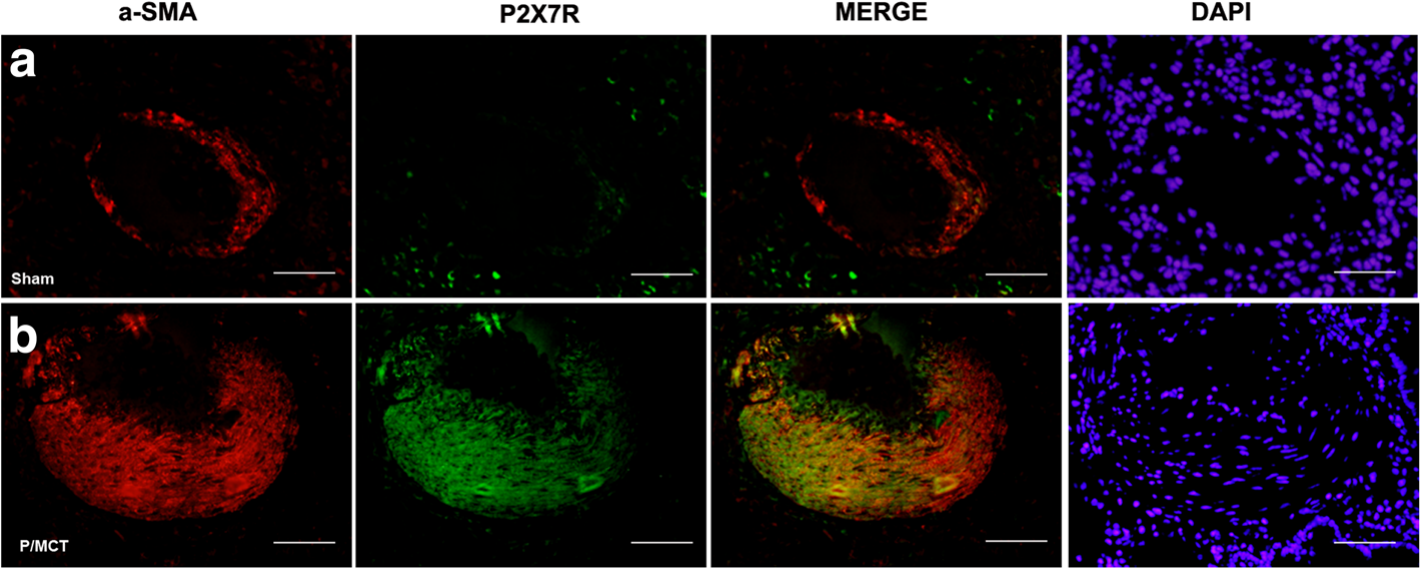
Representative double-immunostained images for P2X_7_R (*Green*), co-stained for α-SMA (*Red*), DAPI (*Blue*) for nuclei and merged images in the sham group (**a**) and P/MCT group (**b**). Original magnification × 40. *Scale bar* = 50 μm for all images

We sought to examine the expression of P2X_7_R in diseased PH lung vessels in rats receiving MCT plus left pneumonectomy, in which the pattern of vascular remodelling resembled the physiology and pathology of human PAH [30]. PH was validated by a significant increase in RVSP at 4 weeks after MCT injection (Additional file 1: Figure S1A). As a consequence of increased RVSP, the vehicle-treated rats also developed significant right ventricular hypertrophy. Progressive increases in RV/ (LV + S) (Additional file 1: Figure S1B) were also observed. These changes were associated with muscularisation and wall thickening of the pulmonary arterioles (Additional file 1: Figure S1C–H). P2X_7_R staining with both a diffuse pattern in the smooth muscle layer and a punctate (indicated by arrow) pattern on the outer wall of the vessel was observed (Fig. 1b and c), while immunoreactivity was restricted to rare inflammatory and lung structural cells and was barely detectable in the pulmonary artery under normal conditions (Fig. 1a), consistent with previous studies [31, 32]. Co-staining of P2X_7_R with a-SMA further confirmed that P2X_7_R was largely distributed in PA-SMCs from the hypertrophied media of pulmonary vessels in PH lung tissue (Fig. 2), suggesting that P2X_7_R might be a novel risk factor contributing to vascular damage.

### Systemic blockade of P2X_7_R inhibits the NLRP3/ IL-1β pathway in rats with MCT-induced PH

**Fig. 3:**
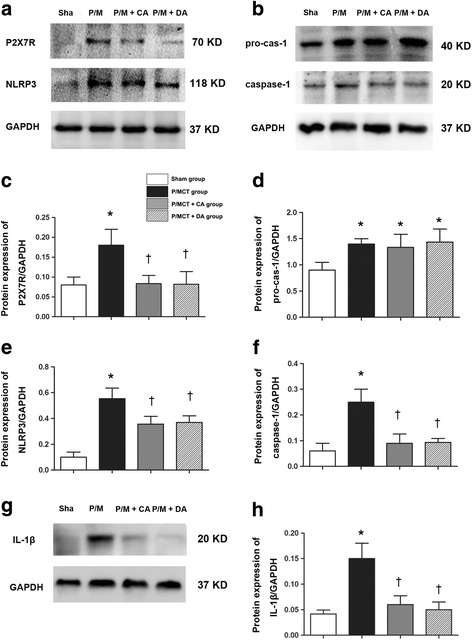
Activation of the P2X_7_R and inflammasome in PH rats. Animals were treated with P/MCT, P/MCT plus early and continuous A740003 (P/MCT + CA), or P/MCT plus delayed A740003 (P/MCT + DA) as described in the METHODS. The levels of P2X_7_R (70 kDa), NLRP3 (118 kDa) (**a**) procaspase-1 (Pro-Casp1, 40 kDa), active caspase-1 (Casp1, 20 kDa) (**b**) and mature IL-1β (20 kDa) (**c**) were measured in total lung homogenates from PH rats 4 weeks after MCT exposure using western *blot*. Quantification of protein expression is shown, respectively (**c**–**f**, **h**); GAPDH was used as a loading control (37 kDa). Data are shown as the mean ± SD, *n* = 6, *p < 0.05 compared with the sham group; †*p* < 0.05 compared with the P/MCT vehicle group

**Fig. 4:**
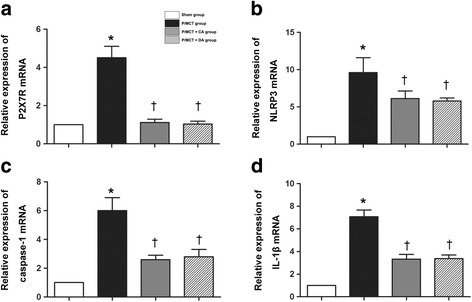
Quantification of mRNA expression of P2X_7_R (**a**), NLRP3 (**b**), caspase-1 (**c**), and IL-1β (**d**) as measured by RT-PCR. Quantitative RT-PCR results represent reactions that were performed in triplicate and normalised to GAPDH expression. The data represent at least three independent experiments. *p < 0.05 compared with the sham group; †*p* < 0.05 compared with the P/MCT vehicle group

We applied the novel A-740003 for P2X_7_R inhibition to explore the role of P2X_7_R in the pathogenesis of PH. We tested the impact of P2X7 inhibition on a recently recognised inflammatory pathway, NLRP3 inflammasome-dependent activation of IL-1β. Western blot and RT-PCR demonstrated that the expression level of NLRP3 (Fig. 3a) and caspase-1 (we analysed the active subunit p20) (Fig. 3b) were upregulated in MCT-treated pneumonectomised rats compared with the sham group, which is inconsistent with previous findings showing NLRP3 inflammasome activation during lung inflammation under the pathologic condition of PH in rats [7]. In PH rats, both treatment and pretreatment with A-740003 efficiently abolished the upregulation of P2X_7_R and greatly decreased the increase in caspase-1 and IL-1β (Fig. 3B–H). Similar results were observed with respect to the mRNA level of P2X_7_/NLRP3 signalling protein (Fig. 4).

### P2X_7_R inhibition suppresses cytokine levels

**Fig. 5:**
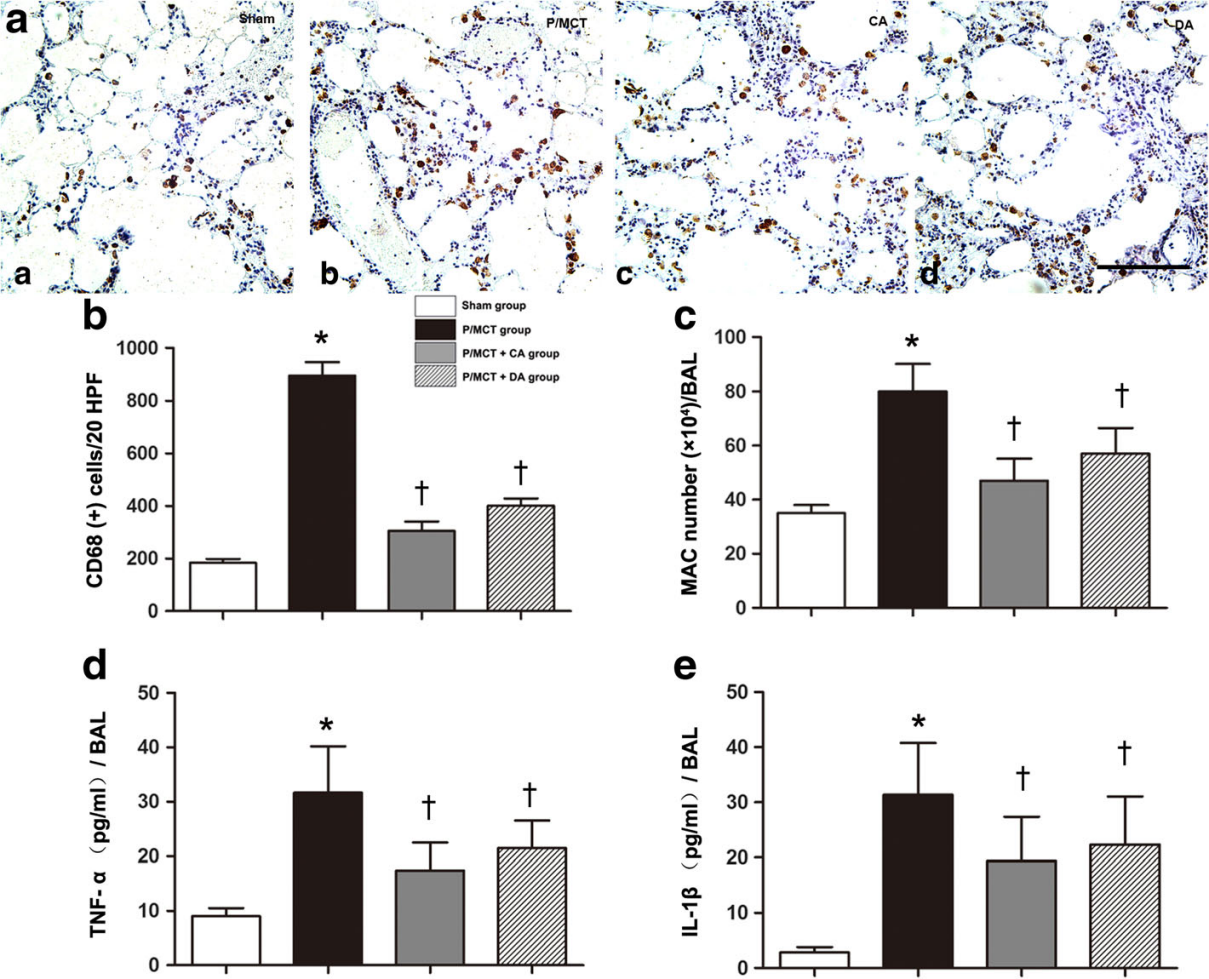
Effect of A-740003 on inflammatory status. **a**, **b** Immunohistochemical staining of macrophages with CD68 antibody in the (*a*) sham, (*b*) P/MCT vehicle group, (*c*) P/MCT + CA group, and (*d*) P/MCT + DA group and the numbers of CD68-positive macrophage cells per 20 high-power fields (HPFs). **c** Macrophage cell count in the bronchoalveolar lavage (BAL). **d** Tumour necrosis factor-α (TNF-α) and **e** interleukin-1β (IL-1β) in the BALF of each treatment group. Original magnification × 40. *Scale bar* = 50 μm for all images. *p < 0.05, **p < 0.01 compared with the sham; †*p* < 0.05 compared with the P/MCT vehicle group

Inflammatory cell recruitment is a key feature in the development of PAH. P2X7R is critical for macrophage infiltration and activation in lung infectious disease [33]. Co-staining of P2X_7_R and CD68 suggested an activation of the P2X_7_R biosynthetic machinery in macrophages (Additional file 1: Figure S2). Of note is CD68, which is an important macrophage marker. Quantification of CD68-positive macrophages by IHC analysis revealed significantly reduced macrophage infiltration both by treatment and pretreatment with A-740003 (Fig. 5a-b). Moreover, BAL samples from P/MCT animals demonstrated a large increase in the number of macrophages after monocrotaline challenge; in contrast, treatment and pretreatment with A-740003 largely reduced macrophage infiltration in the BAL (Fig. 5c) compared with vehicle control animals. Furthermore, pro-inflammatory cytokines, especially TNF-α and IL-1β levels in lavage detected by an ELISA, were significantly reduced in rats treated with A-740003 compared with those in vehicle controls (Fig. 5d and e).

### A-740003 ameliorates pulmonary hypertension

**Fig. 6:**
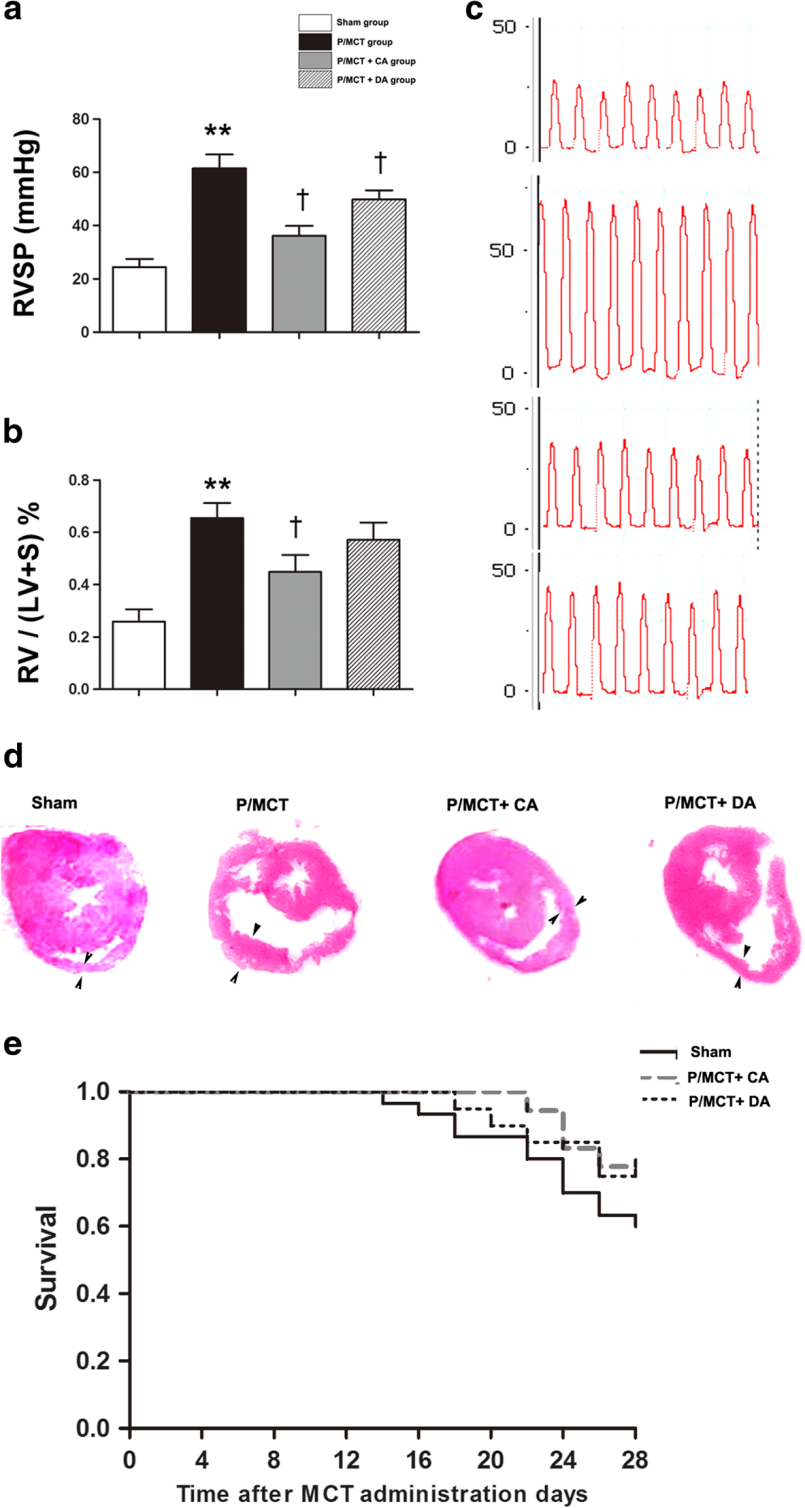
Measurement of right ventricular systolic pressure (using a Millar catheter) shows a reduction in the RVSP in PH rats following pretreatment and treatment of the animals with A-740003 (**a**, **c**). A-740003 also reduced the RV/LV + S ratio in the MCT-treated rats (**b**). The representative visual shape of the RV is also presented (**d**). Kaplan-Meier survival *curves* show that A-740003-treated rats had a non-significantly higher survival rate than those in the vehicle group (**e**). The data are presented as the mean ± SD. *n* = 15–18. **p < 0.01 compared with the *sham group*; †*p* < 0.05 compared with the *P/MCT vehicle group*. RVSP, *right ventricle* systolic pressure; RV/LV + S ratio, weight ratio of the *right ventricle* to the *left ventricle* plus the septum

Next, we analysed the haemodynamic changes at 4 weeks to determine whether P2X_7_R inhibition affects RV pressure and right heart hypertrophy. As a result, RVSP was attenuated in rats that were pretreated with A-740003 (33.6 ± 2.9 mmHg, *p* < 0.05 vs. the P/MCT group) (Fig. 6a and c).

The ratio of the right ventricular weight to the sum of the left ventricular and septum weights (RV/[LV + S]) increased 3-fold in vehicle-treated rats relative to the ratio observed in sham control rats (Fig. 6b). In contrast, pretreatment with A-740003 significantly reversed the elevated right ventricular systolic pressure and the RV/(LV + S) and RV/BW ratios (*p* < 0.05, Fig. 6b and *p* < 0.05, Table 1, respectively). While administration of A-740003 after MCT injection significantly decreased RVSP, it remained higher than that in the A-740003 pretreatment group. However, no further significant improvement in RV hypertrophy was observed (*p* >0.05) (Fig. 6b), perhaps due to the smaller decrease in RVSP and the short observation period.

**Table 1:** Hemodynamics and echocardiography data at 28 day after MCT injection

Parameters	Sham	Vehicle	P/MCT + CA	P/MCT + DA
No. of surviving rats	15	18	16	15
Body weight, g	458 ± 8	390 ± 6*	436 ± 7*^,†^	428 ± 7*^,†^
Heart Rate, bpm	432 ± 9	446 ± 15	440 ± 10	437 ± 17
LV Cardiac Output (ml/min)	145 ± 3	105 ± 4*	107 ± 4*	116 ± 3.4*
RV Ejection Fraction (%)	68.3 ± 3.6	48.2 ± 4.4*	65.6 ± 4.2^†^	62.5 ± 5.1^†^
Pulmonary artery acceleration time, ms	34.2 ± 1	25.8 ± 1.4*	33 ± 0.9†	30.6 ± 0.7^†^
Pulmonary artery, cm	0.32 ± 0.01	0.43 ± 0.04*	0.34 ± 0.02^†^	0.35 ± 0.03^†^
Mean blood pressure	97.3 ± 4.2	90.5 ± 3.8	92.3 ± 2.2	89.4 ± 3.3
RV/BW (mg/g)	3.8 ± 0.2	12 ± 1*	6.2 ± 0.8*^,†^	7.1 ± 0.9*^,†^
RV wall thickness, cm	0.14 ± 0.02	0.23 ± 0.03*	0.16 ± 0.04^†^	0.18 ± 0.02^†^
RV area, mm^2^	12.6 ± 0.4	26 ± 0.8*	14.2 ± 0.5^†^	15.6 ± 0.4^†^
LV area, mm^2^	23.8 ± 0.4	24.6 ± 0.6	24.3 ± 0.7	25.8 ± 0.4

All values are mean ± SD

*Abbreviations*: *RV* right ventricle, *LV* left ventricle, *BW* body weight

*p <0.05 compared with sham group

^†^ *p* < 0.05 compared with respective PAH vehicle group

As shown in Table 1, the rats that received the A-740003 treatment prior to or after MCT administration showed significant reductions in RV wall thickness, RV area, and pulmonary artery diameter compared with the PH rats. We also observed an increased mean acceleration time of the pulmonary artery in the A-740003 treatment group compared with the P/MCT vehicle group, which correlated with a decreased pulmonary pressure. In addition, cardiac output was slightly but not significantly increased in these animals relative to the controls (*p* > 0.05) due to an enhanced stroke volume while the heart rate remained unaltered. Furthermore, the LV area in the PH rats did not significantly change compared with the sham rats. A lower death rate was observed in the A-740003-treated PH rats, although this difference did not reach statistical significance.

### A-740003 reduces pulmonary vascular remodelling

**Fig. 7:**
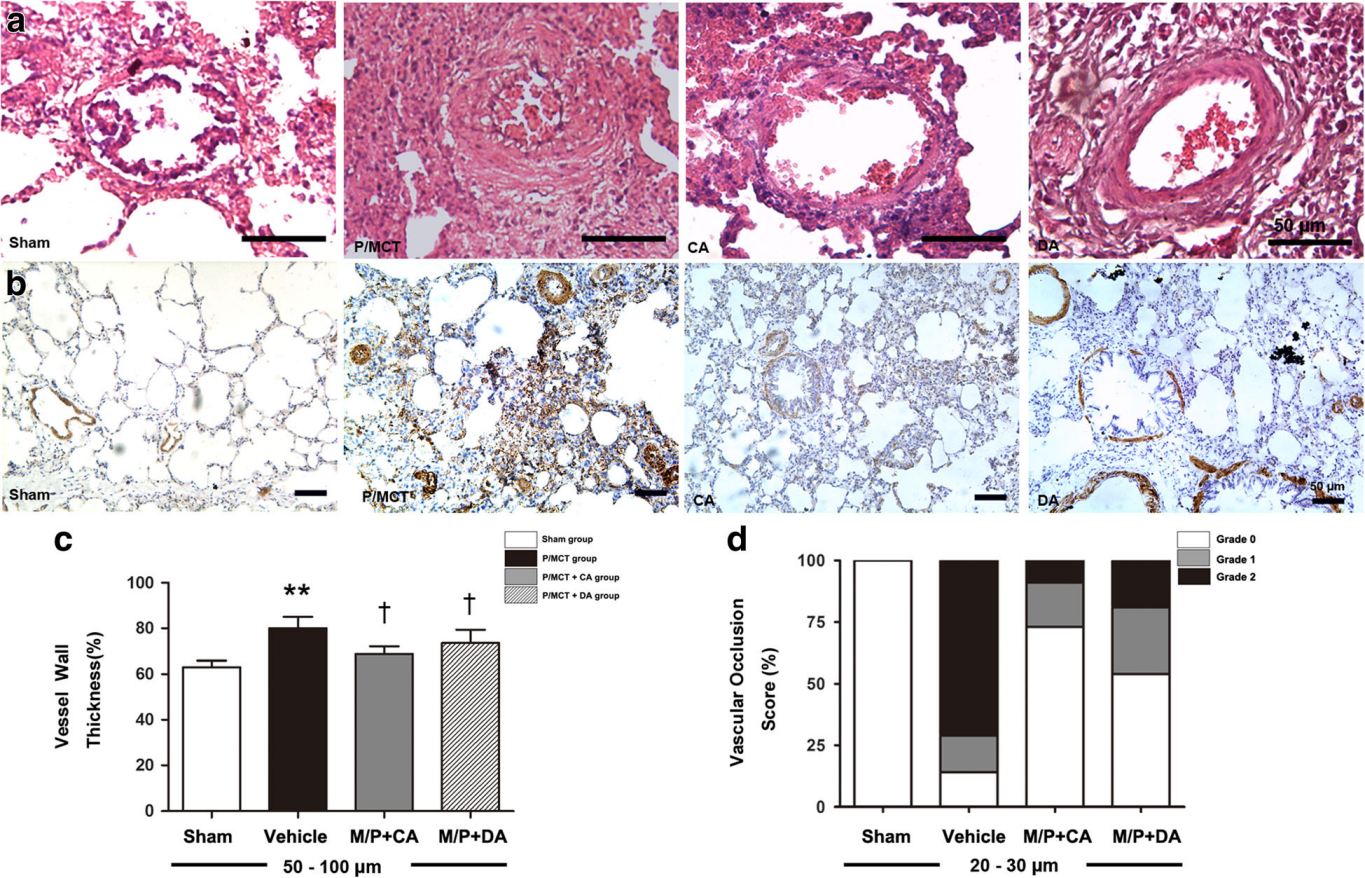
A-740003 ameliorated pulmonary angioproliferation and the development of severe PH. **a** Haematoxylin and eosin and IHC staining of **b** alpha-actin with α-SMA antibody. **c** Ratios of vascular medial thickness (i.e., smooth muscle thickness) to the outer diameter (total vessel wall thickness) of the small pulmonary arteries (diameter 50–100 μm) in PH and normal rats. **d** Vascular occlusion score of the small pulmonary arteries (diameter 20–30 μm) in PH and normal rats. Data are the mean ± SD, *n* = 6. *p < 0.05, **p < 0.01 compared with the *sham group*; †*p* < 0.05 compared with the *P/MCT vehicle group*

PAH causes pulmonary vascular remodelling [34]. A-740003 was administered to animals receiving MCT plus pneumonectomy to determine whether P2X7 inhibition could suppress pulmonary vascular remodelling. We then evaluated the remodelling by measuring the wall thickness and occlusion score of the pulmonary arterioles. As shown in Fig. 7, in vessels with diameters ranging from 50 to 100 μm, wall thickness significantly increased from 63.1% ± 2.6% (sham group) to 79.8% ± 4.5% (*p* < 0.05 vs. sham group). Both continuous and delayed treatment with A-740003 reduced the IPA wall thickness to 68.8% ± 3.1% and 73.5% ± 5.2%, respectively (*p* < 0.05 vs. P/MCT-control; Fig. 7a and c). Decreases in Grade I and II occlusion were also observed (15 and 71% in the P/MCT group vs. 18 and 9% in the P/MCT+ CA group, 27 and 19% in the P/MCT+ DA group, respectively; Fig. 7b and d).

The downregulated α-SMA expression and reduced intrapulmonary artery (IPA) medial wall thickness and vascular occlusion scores as a result of P2X_7_R inhibition suggested that A-740003 decreased the severity of pulmonary vascular muscularisation and reversed the progression of pulmonary vascular remodelling.

The proliferation rates of SMCs were measured by immunofluorescence staining with anti-PCNA antibody.

**Fig. 8:**
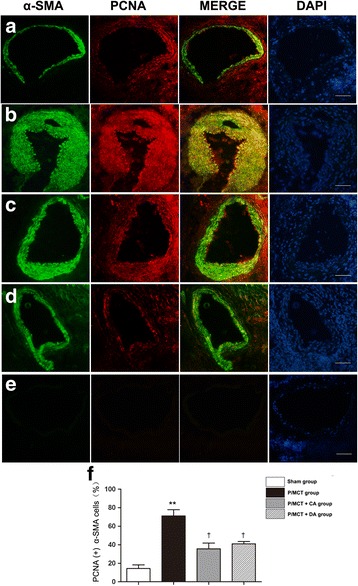
A-740003 decreased the expression of proliferating cell nuclear antigen (PCNA). Immunofluorescence co-staining of PCNA (*Red*) with α-SMA (*Green*) in the **a** sham, **b** P/MCT vehicle group, **c** P/MCT + CA group, **d** P/MCT + DA group and **e** negative control. **f** The mean percentage of PCNA-positive α-SMA cells (magnification × 40, *bar* = 50 μm). Data are the mean ± SD, *n* = 6. **p < 0.01 compared with the *sham group*; †*p* < 0.05 compared with the *P/MCT vehicle group*

**Fig. 9:**
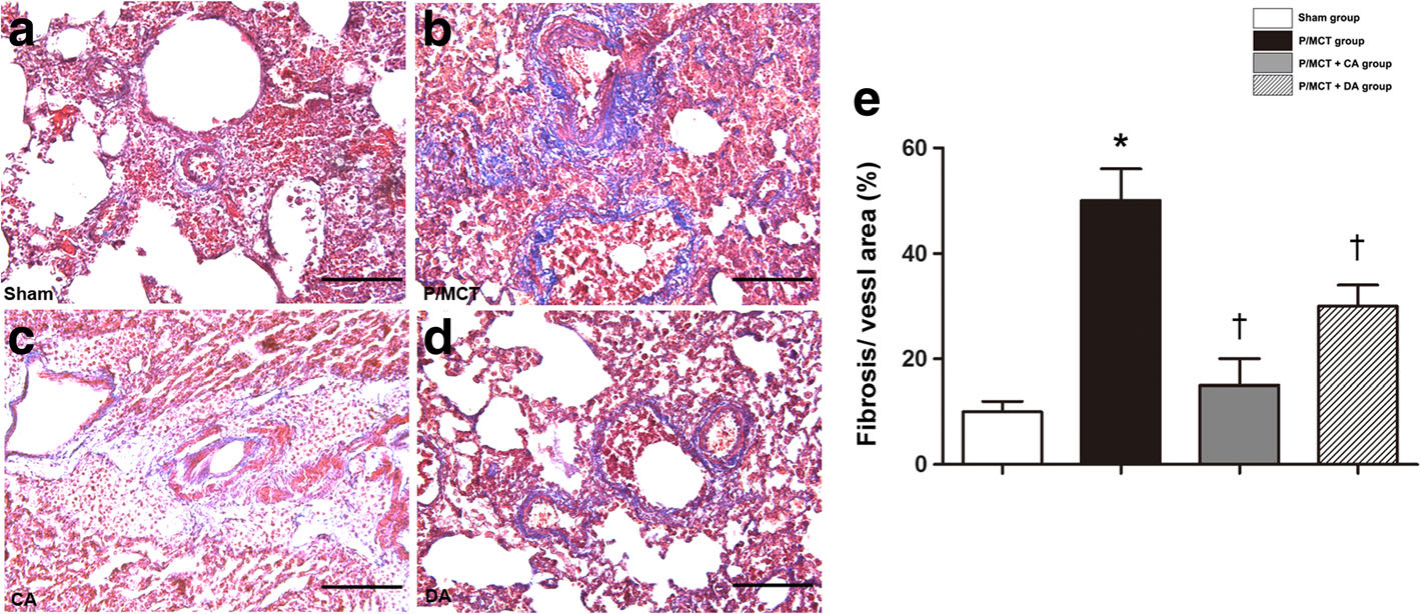
A-740003 prevented lung fibrosis in pulmonary arterial hypertension. Masson trichrome staining for collagen in the lung tissue of the *sham group* (**a**), P/MCT group (**b**), P/MCT + CA group (**c**), and P/MCT + DA group (**d**). Original magnification × 20. *Scale bar* = 50 μm for all images. All values are the mean ± SD, *n* = 6. *p < 0.05 compared with the *sham group*; †*P* < 0.05 compared with the *P/MCT vehicle group*

The mean rate of proliferation (percentage of PCNA-positive a-SMA cells) in the A-740003 group was significantly decreased compared to that in the vehicle-PH group (Fig. 8). Furthermore, Masson trichrome staining showed marked decreases in collagen deposition in the lungs of animals treated with A-740003 relative to the vehicle-treated controls (Fig. 9). These results indicate that P2X_7_R upregulation is involved in MCT-induced pulmonary vascular remodelling. Therefore, remodelling was greatly reduced in the absence of P2X_7_R signalling, outlining a critical role of the P2X_7_R axis in the establishment of lung artery hypertension. These findings demonstrate that inhibition of P2X_7_R decreases RVSP and RVH. These findings indicate that the increased P2X_7_R expression plays an important role in the pathogenesis of MCT-induced PH and RV dysfunction and provide a potential therapeutic target.

## Discussion

To our knowledge, this is the first study to demonstrate the role of P2X_7_R in the pathogenesis of PAH. Pretreatment with A-740003, a P2X_7_R inhibitor, attenuated the development and progression of MCT-induced pulmonary hypertension, RV hypertrophy, and pulmonary arterial neointimal formation in pneumonectomised rats. Moreover, administration of A-740003 even at 2 weeks after MCT could retard the progression of pulmonary hypertension.

Considerable circumstantial evidence suggests that inflammation plays an important role in the pathobiology of PAH. In clinical work, subjects with idiopathic or heritable PAH with higher levels of inflammatory cytokines are associated with higher mortality in PAH [35]. In response to injury and stress, lung vascular cells produce inflammatory mediators, thereby recruiting monocytes/macrophages. Inflammatory cells might continue the release of cytokines and growth factors, forming positive feedback loops. These are characteristic features of the pulmonary inflammatory process and could lead to vascular remodelling by matrix remodelling, collagen deposition, and vascular cell proliferation and migration in PAH. Therefore, the control of inflammation is important for the prevention or treatment of PAH.

In this study, MCT-challenged left pneumonectomised rats showed a marked increase in the recruitment of macrophages into perivascular and peri-alveolar areas of pulmonary tissues and bronchoalveolar lavage samples, consistent with previous reports [36]. Studies have shown that the extracellular P2X_7_R axis contributes to the development of lung inflammation [37], as well as the proliferation and migration of vascular smooth muscle cells [38]. The above data provide evidence for a potential role of P2X_7_R signalling in the development of PAH. We observed increased P2X_7_R activation in the lung tissues of PH rats, most likely due to the early signal of ATP release secondary to stretch-activated channels caused by the elevated pulmonary pressure and MCT-induced lung EC injury [39, 40]. Activated P2X_7_R co-localised with both myeloid lineage cells and constitutive vascular cells. Therefore, we further explored the extent to which P2X_7_R participated in the process of pulmonary vessel remodelling by P2X_7_R inhibitor—A-740003, which is broadly used in preclinical disease models [12]. Our data showed that either pretreatment or treatment with A-740003 could decrease macrophage recruitment. Furthermore, the downregulated expression of pro-inflammatory cytokines identified by ELISA validated the suppressive effect of P2X_7_R-targeted therapy on inflammation in PAH, demonstrating a direct link between P2X_7_R and a pro-inflammatory phenotype during the inflammatory process of PAH. Eventually, A-740003 ameliorated pulmonary vascular remodelling and pulmonary hypertension in both preventive and therapeutic manners. However, the exact mechanism by which A-740003 prevented PH and inflammation remained elusive until the discovery of the NLRP3 inflammasome/IL-1β pathway.

P2X_7_R activation acts as the second signal in NLRP3 inflammasome formation, which results in the cleavage of procaspase-1 into active caspase-1 [41]. Caspase-1 could further cleave the IL-1β precursor to form the mature and secreted forms [42]. P2X_7_R is highly expressed in mononuclear macrophage cell lines. Moreover, most of the pharmacological evidence regarding the therapeutic potential of targeting P2X_7_R was focused on the processing and release of IL-1β from activated monocytes/macrophages and microglia [43–45]. Hence, P2X_7_ may orchestrate macrophage-dominated inflammation during the PH process. In our study, activated P2X_7_R highly co-localised with CD68 positive macrophages in the lung tissue of PH rats. The blockade of P2X_7_ was associated with the inhibition of NLRP3 activation and the decrease of mature IL-1β release, compatible with a scenario in which sustained P2X_7_R activation of alveolar macrophages somehow provided the second signal necessary for NLRP3/ASC/caspase-1 inflammasome aggregation, proteolytic maturation/activation of caspase-1, pro-IL-1β cleavage, and subsequent IL-1β release. The mature IL-1β is a prototypic multifunctional cytokine that is involved in pulmonary inflammation and could stimulate chemokines and adhesion molecules, such as MCP-1 and MIP-1α for macrophage recruitment [46, 47]. Therefore, the mechanisms responsible for the decrease in macrophage infiltration by A-740003 may be caused by a specific decrease in IL-1β. Both caspase-1 and IL-1β have been shown to participate in the pathogenesis of experimental PH in gene knockout and specific antagonist studies [8, 48, 49]. Therefore, P2X_7_R potentially has roles in PH by mediating NLRP3 inflammasome activation and IL-1β secretion. In contrast, A-740003 likely decreased IL-1β production due to the loss of macrophage infiltration and caspase-1 activation, participating in the reversal of PH. We speculate that ATP release acts as an early signal via P2X_7_R activation to trigger pulmonary cell responses including inflammasome activation, leading to mature IL-1β and factor expression remodelling.

Despite being the most important pathophysiological response triggered by the activation of the P2X_7_R/NLRP3 inflammasome axis and release of IL-1β, we showed that P2X_7_R was also strongly expressed by PA-SMCs. Therefore, we could not rule out the possibility that P2X_7_ may function on PA-SMCs directly instead of through the NLRP3/IL-1β pathway. The complex vascular lesions associated with PAH appear to be governed by the same traits that control cancer growth, the absence of apoptotic cells and the presence of anti-apoptotic proteins in the lesion cells. Recently, it has been clearly shown that P2X_7_R can also support cell growth, mainly by increasing the endoplasmic reticulum Ca^2+^ content and the mitochondrial potential, thereby activating NFATc1, preventing apoptosis and promoting cell proliferation [50, 51]. In addition, P2X_7_R has a clear role in the activation of immune cells, and P2X_7_R also mediates the release of factors that can modulate the inflammatory state of vessel wall P2 receptors, which are potential targets for the treatment of hypertension [52]. Histological and molecular analysis revealed that A-740003 treatment abrogated significant ECM deposition (collagens) and PA remodelling in the lungs, supporting the dual effect of P2X_7_R on macrophage migration and activation as well as on PA-SMC proliferation. Does P2X_7_R activation exert direct proliferative and pro-survival roles for the pulmonary artery? To answer this question, future studies focusing on the effects of vascular P2X_7_R signalling on ECs and SMA proliferation should be conducted.

### Clinical implications

Our study proposes that P2X_7_R-specific inhibitors may be a promising approach to improve the life span and quality of life for patients with idiopathic PAH (iPAH). In addition to iPAH, CTD-associated PAH accounts for nearly half of patient disease aetiologies in the oldest age group, which benefits less from iPAH-targeted therapy [53]. Therapy employing the blockade of P2X_7_R pathways has been proven to be effective in various connective tissue diseases (CTDs) [54]. Therefore, A-740003-directed therapeutic strategies also seem to be justified in other forms of severe PAH. The poor prognosis of patients afflicted by this disease despite treatment with currently available vasodilator drugs makes the development of new treatment strategies imperative. If vascular remodelling can be developed from redundant mediators, a combination “cocktail” consisting of anti-remodelling agents such as endothelin inhibitors, prostacyclin, and anti-inflammatory agents such as A-740003 may be more beneficial for patients with PAH than any single agent alone [55].

### Limitations

First, our study lacked specific NLRP3 antagonist interference or NLRP3 gene deficiency analysis in animals to confirm the role of NLRP3 in PAH. Second, other PH models like hypoxia or pulmonary artery banding in rodents must be further investigated in the context of anti-P2X_7_R treatment. Third, A-740003 may function as an off-target effect other than acting on P2X_7_R.

## Conclusion

In summary, the results of this study suggest that P2X_7_ may at least partly mediate PA hypertrophy via the NLRP3/caspase-1 pathway, leading to inflammation and vascular remodelling, which contributes to the development of PH. Inhibition of P2X_7_ is a protective factor and therapeutic target for the amelioration of P/MCT-induced PH. Our results suggest that P2X_7_R inhibition could be a novel therapeutic strategy for the treatment of human PAH. These theories provide a strong impetus for ongoing efforts to define the mechanisms of P2X_7_R signalling.

## Additional file

Additional file 1: Figure S1. Validation of the MCT plus - pneumonectomy induced PAH model. (A-B) Rats were given a single intraperitoneal injection of 60 mg/kg MCT one week after left pneumonectomy or vehicle, and RV systolic pressure (A) and RV weight (B) were measured 1, 2, 3, or 4 weeks after MCT challenge. (C–F) H&E staining and a-SMA staining of lung tissue sections at 4 weeks after MCT injection. (G) The % medial wall thickness was calculated as [(medial thickness × 2)/external diameter] × 100. Scale bars  = 50 μm. All data are expressed as mean ± SD. *n* =  6 per group. ***p* < 0.01 and **p* < 0.05. **Figure S2** Representative double-immunostained images of P2X_7_R (Red), co-stained for FITC-CD68, DAPI (Blue) for nuclei and merged images in the P/MCT (A), sham group (B) and negative control (C), Original magnification × 40. Scale bar = 50 μm for all images. (DOCX 1872 kb)

## Abbreviations

ASC: Apoptosis speck-like protein containing a caspase-recruitment domain
LV: Left ventricle
MCT: Monocrotaline
NLRP3: Nod-like receptor family, pyrin domain containing 3
P/MCT: MCT plus pneumonectomy
P2X_7_R: P2X_7_ receptor
PAH: Pulmonary artery hypertension
RV: Right ventricle
