# Supplementary material for: Role of P2X7R in the development and progression of pulmonary hypertension
Source: Respir Res. 2017 Jun 24;18:127. doi: 10.1186/s12931-017-0603-0 (PMC5483271; doi:10.1186/s12931-017-0603-0)
Supplement: Additional file 1: Figure S1. — Validation of the MCT plus - pneumonectomy induced PAH model. (A-B) Rats were given a single intraperitoneal injection of 60 mg/kg MCT one week after left pneumonectomy or vehicle, and RV systolic pressure (A) and RV weight (B) were measured 1, 2, 3, or 4 weeks after MCT challenge. (C–F) H&E staining and a-SMA staining of lung tissue sections at 4 weeks after MCT injection. (G) The % medial wall thickness was calculated as [(medial thickness × 2)/external diameter] × 100. Scale bars = 50 μm. All data are expressed as mean ± SD. n = 6 per group. **p < 0.01 and *p < 0.05. Figure S2 Representative double-immunostained images of P2X7R (Red), co-stained for FITC-CD68, DAPI (Blue) for nuclei and merged images in the P/MCT (A), sham group (B) and negative control (C), Original magnification × 40. Scale bar = 50 μm for all images. (DOCX 1872 kb) [file 12931_2017_603_MOESM1_ESM.docx]

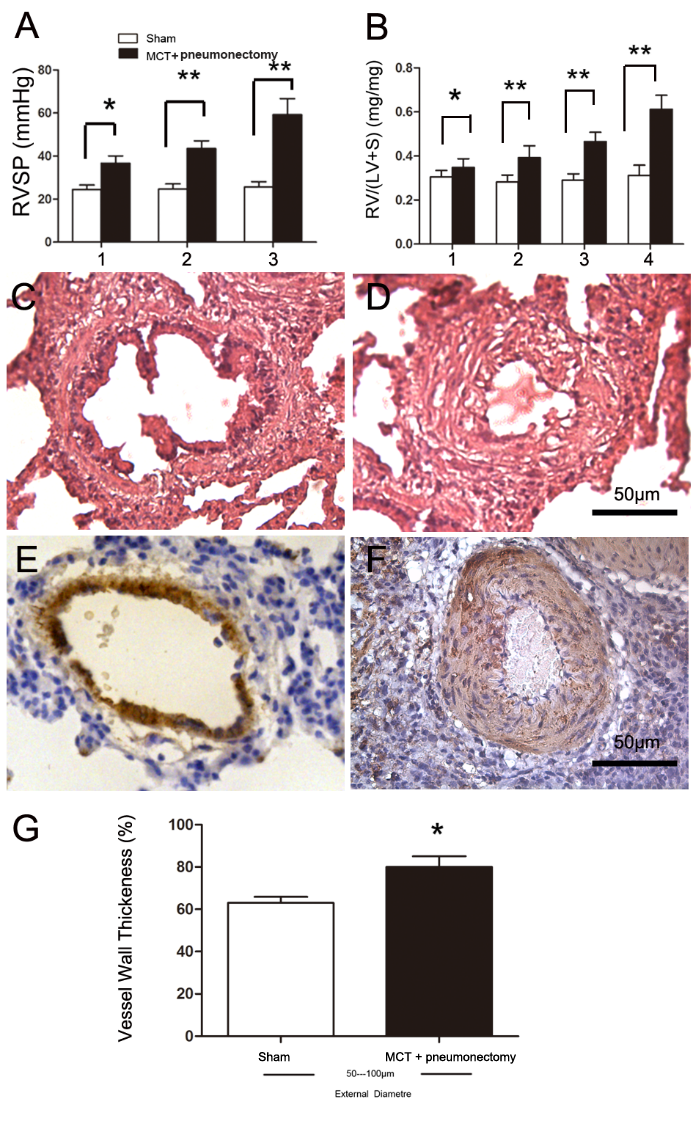


**Figure S1 Validation of the MCT plus - pneumonectomy induced PAH model.** (A–B) Rats were given a single intraperitoneal injection of 60 mg/kg MCT one week after left pneumonectomy or vehicle, and RV systolic pressure (A) and RV weight (B) were measured 1, 2, 3, or 4 weeks after MCT challenge. (C–F) H&E staining and a-SMA staining of lung tissue sections at 4 weeks after MCT injection. (G) The % medial wall thickness was calculated as [(medial thickness×2)/external diameter]×100. Scale bars = 50 µm. All data are expressed as mean ± SD. n = 6 per group. **p<0.01 and *p<0.05.


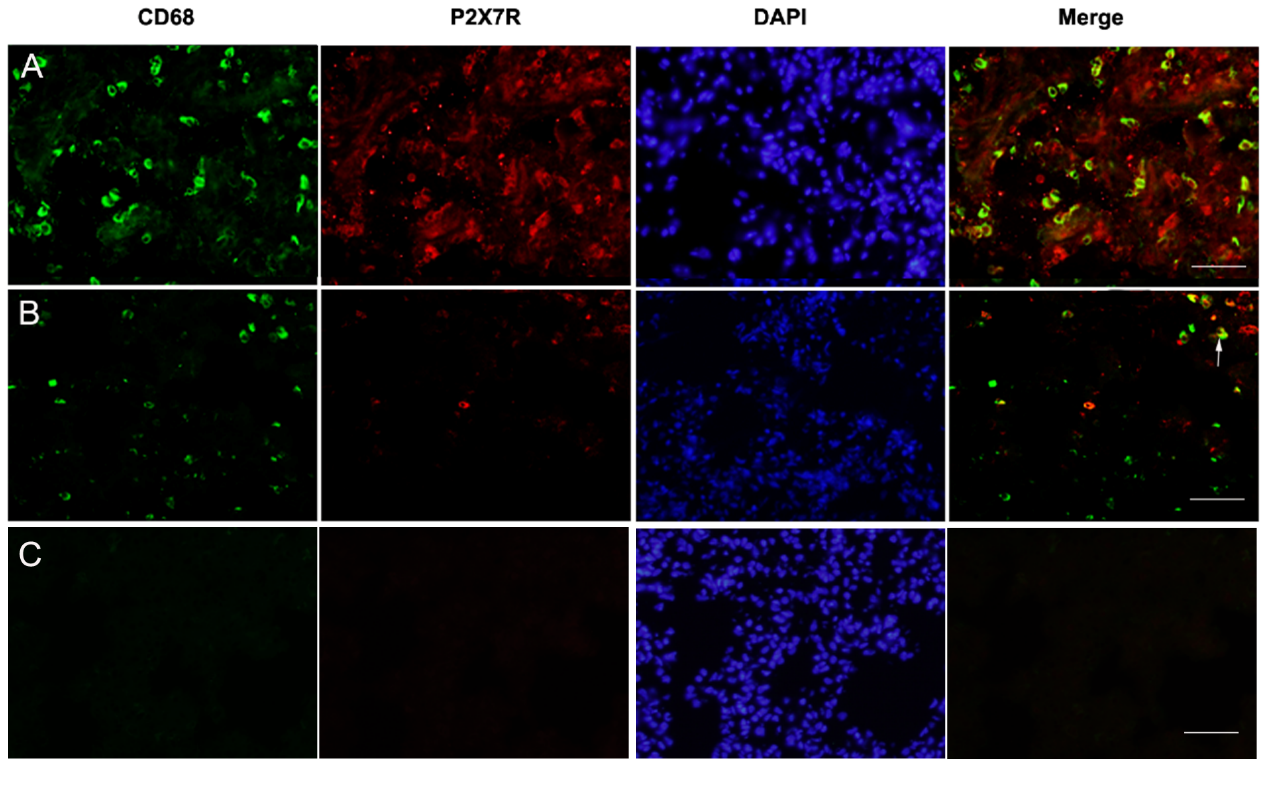


**Figure S2 Representative double-immunostained images** of P2X_7_R (Red), co-stained for FITC-CD68, DAPI (Blue) for nuclei and merged images in the P/MCT (A), sham group (B) and negative control (C), Original magnification × 40. Scale bar = 50 μm for all images.
